# Vestibular, Central, and Non-Vestibular Etiologies of Vertigo and Disequilibrium: A Rural Hospital-Based Cross-Sectional Comparative Analysis

**DOI:** 10.7759/cureus.36262

**Published:** 2023-03-16

**Authors:** Vaidehi Hande, Shraddha Jain, Aditya Ranjan, Mithula Murali, Chandra Veer Singh, Prasad Deshmukh, Sagar S Gaurkar, Smriti Wadhwa, Nimisha Patil, Neha Phate, Venkat Reddy

**Affiliations:** 1 Otolaryngology - Head and Neck Surgery, Jawaharlal Nehru Medical College, Datta Meghe Institute of Higher Education and Research (Deemed to be University), Wardha, IND; 2 Otolaryngology - Head and Neck Surgery, Jawaharlal Nehru Medical College, Datta Meghe Institute of Higher Education and Research (Deemed to be university), Wardha, IND; 3 Department of Medicine, Jawaharlal Nehru Medical College, Datta Meghe Institute of Higher Education and Research (Deemed to be University), Wardha, IND

**Keywords:** proprioceptive cervicogenic vertigo, cervicogenic giddiness, bppv, meniere’s disease, vertigo

## Abstract

Introduction: Vertigo/dizziness is defined as disturbed postural awareness and could range from a feeling of sensation of spinning of self or surrounding. Dizziness or disturbed postural awareness is a common presentation in varying age groups. Vertigo has varied clinical presentations. Classically, there are four vertigo syndromes: vertigo, imbalance/disequilibrium, presyncope/lightheadedness, and psychogenic dizziness. The present study was conducted to examine the various etiologies involved in these syndromes and to help unmask the overlaps between them. This study also aimed to further classify the etiologies underlying these vertigo syndromes and overlaps into peripheral or vestibular, central, and non-vestibular. This would help develop a comprehensive management protocol for vertigo of any origin.

Methods: A prospective observational cross-sectional study was undertaken in a rural hospital in Central India. We studied patients with giddiness and categorized them into vertigo syndromes according to the site of origin of vertigo. We also compared overlaps in the presentation of vertigo.

Results: Out of the 80 patients that were studied, vertigo with disequilibrium was observed in 72.50% of the patients. Non-vestibular vertigo of cervicogenic origin was the common cause of vertigo seen in 36.25% of the patients occurring alone or in association with vestibular vertigo. Among patients with overlaps, vestibular vertigo with non-vestibular vertigo was the most common etiology observed in 89.65% of the patients with overlaps.

Conclusion: The syndrome of “vertigo with disequilibrium” was the commonest presentation in the patients studied, followed by “vertigo syndrome” as an isolated symptom, not associated with “disequilibrium.” *Ours is probably the first study to report this observation of overlaps of two syndromes, with diagnostic implications.*

## Introduction

Vertigo/dizziness is defined as disturbed postural awareness and could range from a feeling of sensation of spinning of self or surrounding. Dizziness or disturbed postural awareness is a common presentation, occurring in various age groups and expressed differently by the patients. Classically, there are four vertigo syndromes: vertigo, imbalance/disequilibrium, presyncope/light-headedness, and psychogenic dizziness [1]. It is a challenge for otorhinolaryngologists to diagnose aetiologies of vertigo with different presentations, which could be the result of a multitude of etiologies, ranging from those located at different sites in the vestibular pathway, and their connections. Opinion from neurologists, neurosurgeons, orthopedics, and internal medicine physicians is often required. 

Cervicogenic vertigo as an etiology of vertigo syndromes and disequilibrium has been mentioned by a few authors but has not received universal acceptance with no mention in otolaryngology textbooks [2-4]. Vertigo refers to a sensation of movement that may be perceived as own self-moving with respect to the surrounding or as surrounding moving with respect to the person and is the most common type. It may be associated with head movements and nystagmus. The causes can be attributable to the vestibular labyrinth (Meniere’s disease, benign paroxysmal positional vertigo (BPPV), labyrinthitis), some conditions of the central nervous system like posterior circulation stroke and vertebrobasilar insufficiency, and cervicogenic giddiness (Barré-Lieou syndrome and rotational vertebral artery occlusion). Disequilibrium or imbalance is a sensation of falling down mostly while walking. Patients indicate that they are dizzy but “not at the head” and prefer sitting or lying down [5]. It is usually seen in neurological disorders like Parkinson’s disease and peripheral neuropathies. Medications like benzodiazepines and tricyclic antidepressants may also cause disequilibrium [2]. Cerebellar and spinal cord pathologies can also cause such symptoms [3]. There may be associated symptoms like weakness, ataxia, or memory loss. Proprioceptive cervical vertigo (PCV) may also cause disequilibrium [6]. Presyncope or light-headedness or blackouts may be described as a feeling of fainting lasting for a few seconds. It can occur on getting up from a lying position in patients with orthostatic hypotension or on some medications. Most cases represent underlying cardiovascular conditions [1,7]. It is believed to be vestibular in some cases but not confirmed by classic vestibular function tests [8]. Some patients may not be able to describe their symptoms and may give vague complaints most of the time due to psychiatric disorders [1,5]. Psychiatric causes are the second most common type, accounting for 15% of patients with giddiness [5,8].

Cervicogenic giddiness as a cause of imbalance has not received attention. The authors of the present study noted that cervicogenic dizziness, a form of non-vestibular vertigo, can coexist with vestibular syndromes in a majority of cases (e.g., cervicogenic dizziness and Meniere’s disease or BPPV) [2]. The present study was conducted to examine the various etiologies involved in these syndromes and to help unmask the overlaps between them. The study also aimed to further classify the etiologies underlying these vertigo syndromes and overlaps into peripheral or vestibular, central, and non-vestibular. This would help develop a comprehensive management protocol for vertigo of any origin in order to avoid unnecessary investigations and associated costs and delays in diagnosis.

## Materials and methods

This was a prospective cross-sectional observational study conducted in the department of otorhinolaryngology located at a rural teaching hospital in Central India from December 2020 to December 2022 after approval from the institutional ethics committee (Ref No. DMIMS(DU/IEC/2020-21/9329). The sample size formula was calculated using n = (Z alpha/2 square X P (1-P))/d square, where Z alpha/2 is the level of significance at 5% (1.96) and P is the prevalence of vertigo (0.71% or 0.071). A minimum of 75 patients were studied.

**Figure 1:**
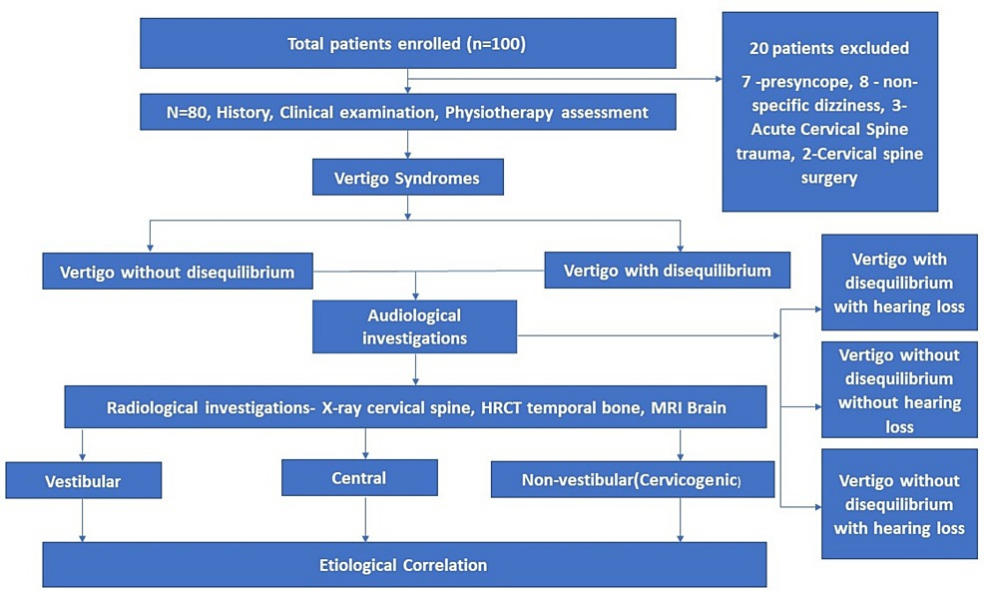
Methodology followed for the study HRCT: High-resolution computed tomography

All the patients that presented with vertigo and giddiness in the otolaryngology outpatient department or emergency or were referred from other specialties like general medicine, neurology, neurosurgery, orthopedics, gynecology, and pediatrics were included in this study. The following were excluded from the study: The vertigo syndromes of presyncope and non-specific giddiness, children below the age group of 10 years, marked cervical spine disc protrusion, history of active inflammatory joint disease, cervical spine cancer or infection, bone diseases or marked osteoporosis, spinal cord pathology, fracture or dislocation of the neck, acute cervical nerve root symptoms, and previous surgery to the upper cervical spine. The detailed methodology followed for this study is depicted in Figure 1.

Out of the 100 patients who presented with vertigo and giddiness in the otolaryngology outpatient department or emergency or were referred from other specialties like general medicine, neurology, neurosurgery, orthopedics, gynecology, and pediatrics were included in this study after obtaining written informed consent. The diagnosis of the patients was made mostly on clinical grounds. The patients were categorized into vertigo syndromes: (1) vertigo, referring to a sensation of movement which may be perceived as own self-moving with respect to the surrounding or as surrounding moving with respect to the person, (2) disequilibrium, a sensation of falling down mostly while walking, (3) pre-syncope, described as a feeling of fainting and may be associated with excessive salivation, heat, or diplopia and may last for seconds and minutes, and (4) non-specific dizziness, not able to describe their symptoms and may give vague complaints most of the time due to psychiatric disorders [1-4]. After applying exclusion criteria, 80 patients with vertigo and disequilibrium were enrolled and subjected to investigations like pure-tone audiometry and further classified into those with and without hearing loss; a high-resolution computed tomography (HRCT) of the temporal bone was done to diagnose vertigo originating from conditions of the temporal bone, an MRI of the brain for central causes of vertigo was done based on history and clinical examination, X-rays of the cervical spine anteroposterior and lateral view was done where cervicogenic etiology was suspected based on the history (neck pain, back pain, unsteadiness, limited range of cervical motion), and an MRI of the cervical spine was done in certain cases where conditions of cervical spine mentioned in exclusion criteria were suspected. The etiologies were further subclassified as vestibular, central, and non-vestibular of cervicogenic origin based on the site of the lesion. The diagnosis of vestibular vertigo was made when the lesion involved semi-circular canals, bony and vestibular labyrinth, and the cranial nerve eight [9]. When the lesion involved the vestibular nuclear complex, vestibulo-cerebellum, brainstem, spinal cord, and vestibular cortex, central vertigo was diagnosed [10]. A diagnosis of non-vestibular vertigo of cervicogenic origin was made when the patient presents with complaints of neck pain, unsteadiness, and limited range of cervical motion [11,12]. Cervical spine X-rays were studied for straightening of the spine, reduced intervertebral disc spaces, over-crowding of cervical vertebrae, and increase in anterior inter-vertebral disc spaces.

Statistical analysis

Statistical analysis was performed using Epi Info^TM^ version 7.2.2.2, a trademark of the Centers for Disease Control and Prevention. A descriptive statistical analysis was performed to calculate the mean and standard deviation. A test of proportion was used to find the standard normal deviation (Z), and the p-value <0.05 was considered statistically significant.

## Results

Baseline characteristics of patients with giddiness

A total of 80 patients were evaluated, and their baseline parameters are depicted in Table 1.

**Table 1:** Baseline characteristics of patients with vertigo SD: Standard deviation

Parameters	Number of patients n=80 (%)
Age groups	
11-30	25 (31.25)
31-50	36 (45)
51-70	19 (23.75)
Mean±SD	40.50±16.04
Gender	
Male	30 (37.50)
Female	50 (62.50)
Occupation	
Farmer	17 (21.25)
Laborer	6 (7.50)
Engineer	13 (16.25)
Clerk	9 (11.25)
Student	12 (15)
Army veteran	2 (2.5)
Housewife	14 (17.50)
Unemployed	4 (5)
Doctor	2 (2.5)

The test of proportion showed that the proportion of females (62.50%) was significantly higher than that of males (37.50%) (Z=3.67;p<0.001). In this study, farmers (21.25%) were more prone to have vertigo syndromes than others (Z=2.61; p=0.009) as depicted in Table 1.

Distribution of patients according to vertigo syndromes

Among the 80 patients, 58 (72.50%) had vertigo with disequilibrium (syndromes with overlaps) (Z= 6.64;p<0.0001) as depicted in Table 2.

**Table 2:** Vertigo syndromes

Vertigo syndromes	No. of patients = n (%)
Vertigo without disequilibrium	22 (27.50)
Vertigo with disequilibrium (syndromes with overlaps)	58 (72.50)
Total	80

Incidence of vertigo without disequilibrium in patients with different etiologies

Out of the 22 patients with vertigo without disequilibrium, six patients were of BPPV. (Out of the 13 patients who presented with BBPV as a cause of vertigo, the rest of the 7 patients had vertigo with disequilibrium and are covered under the next heading.) The various etiologies of vertigo without disequilibrium are depicted in Table 3.

**Table 3:** Etiologies of vertigo without disequilibrium BPPV, benign paroxysmal positional vertigo; COM, chronic otitis media

Sr no	Probable etiologic diagnosis	No. of patients (N) (%)
1	BPPV	06/13 (46.15)
2	Meningioma (early stage)	01/02 (50)
3	Post-traumatic	02/09 (22.22)
4	Meniere’s disease	03/10 (30)
5	COM with labyrinthitis	03/03 (100)
6	Acoustic neuroma	01/02 (50)
7	COM - labyrinthine fistula	01/05 (20)
8	Superior semi-circular Dehiscence syndrome	03/03 (100)
	Total	22 (27.50%)

Incidence of vertigo with disequilibrium in patients with different etiologies

Vertigo with disequilibrium was present in seven out of the ten (70%) patients with Meniere's disease, 17 out of the 17 (100%) patients with PCV, and among a total of 58 patients with vertigo with disequilibrium. The various etiologies of vertigo with disequilibrium are depicted in Table 4.

**Table 4:** Etiologies of vertigo with disequilibrium PCV, proprioceptive cervical vertigo; BPPV, benign paroxysmal positional vertigo; COM, chronic otitis media; CP, cerebellopontine

Probable etiologic diagnosis	No. of patients (%)
Meniere’s disease with PCV	07/10 (70)
BPPV with PCV	07/13 (53.83)
PCV	17/17 (100)
Vestibular migraine	07/07 (100)
Post-traumatic with PCV	05/09 (55.55)
COM-labyrinthine fistula with PCV	04/05 (80)
Rotational vertebral artery vertigo	03/03 (100)
Vestibular neuronitis	02/02 (100)
CP angle meningioma (late stage)	01/02 (50)
Cerebral tumors	01/01 (100)
Acoustic neuroma	01/02 (50)
Barré-Lieou syndrome	01/01 (100)
Posterior circulation stroke	01/02 (50)
Posterior circulation stroke with PCV	01/02 (100)
Total	58

Classification of etiologies of vertigo syndromes based on association with hearing loss

Vertigo without disequilibrium was seen in 100% of the patients with Meniere's disease; vertigo without disequilibrium without hearing loss was present in 46.15% of the patients with BPPV; vertigo with disequilibrium was present in 100% of the patients with Meniere's disease with PCV. The various etiologies of vertigo syndromes and their association with hearing loss are depicted in Table 5.

**Table 5:** Association of hearing loss with vertigo and disequilibrium COM, chronic otitis media; BPPV, benign paroxysmal positional vertigo; PCV, proprioceptive cervical vertigo

Vertigo without disequilibrium with hearing loss (%)	Vertigo without disequilibrium without hearing loss (%)	Vertigo with disequilibrium with hearing loss (%)
Meniere’s disease (100)	BPPV (46.15)	BPPV with PCV (42.85)
COM with labyrinthitis (100)	Post-traumatic (22.22)	Post-traumatic with PCV (44.44)
Acoustic neuroma (early stage) (50)	Meningioma (early stage) (50.00)	COM-labyrinthine fistula with PCV (100)
COM-labyrinthine fistula (20)		Meniere’s disease with PCV (100)
Superior semi-circular dehiscence syndrome (100)		Acoustic neuroma (late stage) (50)
		PCV (38.88)
		Vestibular migraine (14.28)

Site of origin of vertigo

The most common site of origin of vertigo was non-vestibular (cervicogenic type) seen in 36.25% of the patients.

Among the multiple sites of origin of vertigo vestibular vertigo with non-vestibular vertigo (cervicogenic type) was seen in 89.65% of the patients. The site of origin of various etiologies of vertigo is depicted in Table 6.

**Table 6:** Site of origin of vertigo

Site of origin		No. of patients (%)
Vestibular		17 (21.25)
Central		05 (6.25)
Non-vestibular (cervicogenic type)		29 (36.25)
Multiple sites of origin (n=29)	Vestibular with non-vestibular (cervicogenic)	26/29 (89.65)
	Central with non-vestibular (cervicogenic)	03/29 (10.35)
Total		80

Etiologies of vestibular, central, and non-vestibular vertigo

Among the vestibular etiologies of vertigo, the most common was BPPV present in 28.88% of the patients; acoustic neuroma, posterior circulation stroke, and cerebellopontine (CP) angle meningioma each accounted for 28.57% of the patients' cases of central causes of vertigo; PCV was the most common etiology of non-vestibular vertigo seen in 60.71% of the patients. The other etiologies of vestibular, central, and non-vestibular vertigo are depicted in Table 7.

**Table 7:** Etiologies of vestibular, central, and non-vestibular vertigo BPPV, benign paroxysmal positional vertigo; CSOM, chronic suppurative otitis media; CP, cerebellopontine

	No. of patients (%)
Etiologies of vestibular vertigo n=45	
Meniere’s disease	10 (22.22)
BPPV	13 (28.88)
Post-traumatic	09 (20)
Labyrinthitis	3 (6.66)
Vestibular neuronitis	2 (4.44)
Superior semicircular canal dehiscence syndrome	3 (6.66)
CSOM-labyrinthine fistula	5 (11.11)
Etiologies of central vertigo n=7	
Acoustic neuroma	2 (28.57)
Posterior circulation stroke	2 (28.57)
Cerebral tumor	1 (14.28)
CP angle meningioma	2 (28.57)
Etiologies of non-vestibular vertigo n=28	
Barré-Lieou syndrome	01 (3.57)
Rotational vertebral artery vertigo (Bow Hunter's syndrome)	03 (10.71)
Vestibular migraine	07 (25)
Propioceptive cervicogenic vertigo	17 (60.71)

 Coexistence of non-vestibular vertigo with vestibular vertigo

Table 8 depicts the frequency of association of non-vestibular vertigo of cervicogenic origin (PCV category) with vestibular vertigo of various types. The frequency of association of PCV with Meniere's disease was as high as 70% and BPPV was 53.84%.

**Table 8:** Frequency of association of non-vestibular vertigo with various types of vestibular vertigo PCV, proprioceptive cervical vertigo; CSOM, chronic suppurative otitis media; BPPV, benign paroxysmal positional vertigo

Non-vestibular vertigo associated with vestibular etiologies of vertigo	No. of patients (%)
PCV with Meniere’s disease	07 /10 (70)
PCV with post-traumatic vertigo	06/09 (66.66)
PCV with CSOM-labyrinthine fistula	04/05 (80)
PCV with BPPV	07/13 (53.84)

## Discussion

The pathophysiology underlying vertigo or disequilibrium is an imbalance in the vestibular, visual, or proprioceptive inputs. This could be in the form of unilateral or bilateral vestibular hypofunction as in vestibular causes of vertigo; decrease in proprioceptive inputs in sensory ataxias or hypofunction at the level of the cerebellum (central organ of balance) as in central causes of vertigo; or increase in cervical reflexes as in non-vestibular causes (cervicogenic) of vertigo. The cervicospinal, vestibulospinal, reticulospinal, and cervico-colic reflexes are important in maintaining balance. Any abnormalities in these reflex pathways give rise to disequilibrium and vertigo [13]. So, the presentation of vertigo syndromes and the site of localization could be complex.

The present study was conducted on 80 patients of “vertigo with/without disequilibrium” to identify various underlying etiologies and the sites of origin as vestibular, central, and non-vestibular (cervicogenic). “Vertigo without disequilibrium” was seen in 27.50% of the patients, and the majority of the patients (72.50%) presented with “vertigo with disequilibrium.” Post et al. observed that vertigo as a syndrome was present in 45-54% of the patients, and 16% of the patients had disequilibrium [1]. In their series, they did not have overlaps of vertigo with disequilibrium. There is a dearth of literature related to the etiology of overlaps of vertigo syndromes. Ours is probably the first study to report this observation of overlaps of two syndromes, with diagnostic implications.

The syndrome of “disequilibrium alone without vertigo” was not reported in any of our patients, and this was attributed to the fact that disequilibrium is considered a neurological symptom, and, hence, the patients were primarily referred to general physicians, neurologists, or neurosurgeons. Otorhinolaryngology referrals are received only when the patients present with vertigo alone or along with disequilibrium, as vertigo is considered primarily an otorhinolaryngology domain. Our study design aimed to highlight the significant gaps in vertigo management in terms of knowledge of etiologies of vertigo syndromes with resultant inadequate referral systems as a multidisciplinary approach. Cervicogenic vertigo has not received universal acceptance as physiotherapy assessment is not a routine protocol of vertigo test battery among otolaryngologists, physicians, and neurologists [13].

In a study on vertigo syndromes by Kroenke et al., the second most common type of vertigo syndrome after “vertigo” was “psychogenic” [8]. Another study observed the association of cervicogenic vertigo, a form of non-vestibular vertigo, with anxiety and depression [14]. The authors of the present study believe that overlooking the entity of the cervicogenic form of non-vestibular vertigo might miss the appropriate diagnosis in a significant number of patients and make them inappropriately labeled as psychogenic.

As an otolaryngologist who has referrals of patients with vertigo with/without disequilibrium, knowledge of the entity of non-vestibular vertigo of cervicogenic origin becomes crucial. We tried to identify the likely etiologic factors in the patients presenting with vertigo with/without disequilibrium.

Among the patients with vertigo without disequilibrium, the etiologies identified were Meniere’s disease, chronic otitis media (COM) with labyrinthitis, superior semi-circular canal dehiscence syndrome, acoustic neuroma, COM with labyrinthine fistula, BPPV, post-traumatic vertigo, meningioma of early stage, and vestibular neuronitis. Our observations on this were similar to studies by other authors [15].

Meniere’s disease is defined by the presence of episodic vertigo, aural fullness, and tinnitus in the affected ear [16]. In our study, vertigo associated with hearing loss was present in all the cases of Meniere’s disease, as an isolated symptom in 30% of cases, and associated with disequilibrium in as many as 70% of cases. This finding has not been reported in any of the previous studies. This association helped us in the further evaluation of the patients for symptoms and signs related to PCV, and all of the 70 patients had positive findings of PCV. This association was also observed in a previous study by the senior author [2].

Similarly, in our study, vertigo was linked to disequilibrium in 53.84% of vertigo cases and was present in all cases of BPPV as an isolated symptom in 46.15% of cases. This finding has not been reported in any of the previous studies. This association helped us further evaluate the patients for symptoms and signs related to PCV; all of these had positive findings of PCV. In the patients of BPPV who had vertigo alone, hearing loss was not a symptom in any of the patients, and in 42.85% of the patients with associated disequilibrium, hearing loss was an associated finding, which is not a normal feature of BPPV. This observation has not been reported previously as the diagnosis of BPPV is excluded when there is hearing loss [17]. Many authors studied the association of BPPV with sudden sensorineural hearing loss and found that the cause of hearing loss in BPPV could be due to a vascular insult, which is a poor prognostic factor [16]. This needs further evaluation.

Acoustic neuroma

An acoustic neuroma is a tumor arising from a vestibular nerve. However, the main symptom is audiological in the form of tinnitus and hearing loss. The association of vertigo and disequilibrium in acoustic neuroma is variable and depends on the stage of the tumor. Our study observed vertigo in 50% of cases of acoustic neuroma, but it was in the early stage. Nam et al. in a study observed the occurrence of imbalance and vertigo in patients of acoustic neuroma and found that vertigo in patients of acoustic neuroma may be due to its close proximity to the inner ear and diffusion of metabolites leading to cochlear and vestibular damage due to internal acoustic meatus involvement. They also noticed a unique association between the size of the tumor and postural instability, with increased postural sway while the tumor was small, improving with an increase in tumor size and then increasing postural sway again with an even larger tumor compressing the brainstem [18]. Other studies observed that acoustic neuroma patients usually have an apparently normal gait in the early stages [19]. Jethanamest et al. observed that the presence of disequilibrium in acoustic neuroma is a predictive factor for its size growth and that disequilibrium with vertigo is seen only in tumors of larger size [20]. Andersen et al. observed that vertigo might arise due to a sensory mismatch occurring between the vestibular-visual stimuli that are expected and the ones that are perceived [21].

In our study, 100% of the patients with superior semicircular canal dehiscence syndrome had vertigo without disequilibrium; however, in a study by Merchant et al., 20% had vertigo with disequilibrium [22].

Vertigo with disequilibrium was present in 100% of cases of PCV, a form of non-vestibular vertigo. The occurrence of disequilibrium in all the patients of PCV emphasizes the need for the evaluation of vertigo syndromes by a team approach, with the inclusion of physiotherapy assessment as a routine part of the protocol. As per various studies, the diagnosis of PCV is based on a history of vertigo, disequilibrium, and neck pain [6,23]. This was similar to our observations that all cases of PCV in our study had vertigo and neck pain, but its association with disequilibrium was seen in 95.5% of the cases. About 100% of the patients with vestibular migraine, Barré-Lieou syndrome, and rotational vertebral artery vertigo had vertigo with disequilibrium. Schubert et al. reported that the presence of down-beat nystagmus, positional vertigo, and imbalance is a feature of rotational vertebral artery vertigo [24].

The coexistence of PVC with different vestibular vertigo presents with vertigo with disequilibrium. Such confusion in the presentation may mislead the diagnosis. In our study, vertigo with disequilibrium as a symptom was seen in vestibular conditions, when associated with conditions causing non-vestibular vertigo, such as PCV. The conditions diagnosed in our study were all patients of Meniere’s disease associated with PCV and BPPV with PCV in 53.84% of the patients. The senior author observed in a previous study that in patients with neck pain or headache, neck tightness, or asymmetry of the shoulder, vestibular vertigo of MD and BPPV could be a spectrum of the same underlying condition as PCV of non-vestibular type [2]. In our study, vertigo with disequilibrium was seen in all the patients with vestibular neuronitis with PCV. Cooper also observed in his study that vestibular neuronitis was associated with a sudden onset of vertigo, imbalance, and tinnitus but without hearing loss [25].

Central causes of vertigo with disequilibrium were all the patients with posterior circulation stroke and cerebral astrocytoma and 50% of the patients with late-stage CP angle meningioma and acoustic neuroma. An acoustic neuroma in its early stages causes only vertigo with hearing loss, as observed previously in our study. Similar observations were noted in the study by Hunter et al. [26].

In our study, the association of hearing loss with vertigo syndromes was also studied to aid in the diagnosis. Vertigo without disequilibrium, with associated hearing loss, was seen in all 100% of the patients with COM with labyrinthitis, and superior semi-circular canal dehiscence syndrome; 50% of the patients with acoustic neuroma and early-stage meningioma, COM with labyrinthine fistula in 30% of the cases, and post-traumatic labyrinthine concussion in 22.22% of the cases in our study. Similar observations were noted by Brandt et al. in a study of 14,790 patients; however, post-traumatic labyrinthine concussion was not mentioned in their study [15].

Vestibular, central, and non-vestibular vertigo of cervicogenic origin

Purely vestibular vertigo was found in 21.25% of the patients, and purely central vertigo was found in 6.25% of the patients. In our study, out of the 80 patients, 36.25% of the patients, the site of the lesion of vertigo was identified as non-vestibular (of unknown origin likely cervicogenic). Among multiple sites of origin, vestibular with non-vestibular was most commonly present in 89.65% of the patients, whereas the central with the non-vestibular site of origin was found in 10.35% of the patients. None of the previous studies classified vertigo according to the above-mentioned categories. The occurrence of overlaps due to multiple sites of origin of vertigo like non-vestibular with vestibular emphasizes the need for a multidisciplinary approach in vertigo management.

Among the causes of vertigo of vestibular origin, the most common was BPPV and Meniere’s disease. Similar observations were noted by Brandt et al. in a study of 14,790 patients [15]. Studies showed posterior circulation stroke as a common cause of central vertigo [27]. In the present study, the most common cause of non-vestibular vertigo was PCV. In a study by Sunitha et al, vestibular migraine was seen in 3.8% of the patients, and 13.5% of cases had PCV [28].

Association of vestibular with non-vestibular vertigo

The association of non-vestibular cervicogenic vertigo was seen with vestibular vertigo of primary labyrinthine conditions like Meniere's disease and BPPV and secondary involvement of vestibular labyrinth due to infective and traumatic etiology. Kalland Knapstad et al. in their study observed increased morbidity due to the association of neck pain with underlying vertigo [29]. In our study, 70% of the patients with Meniere’s disease of vestibular vertigo were associated with PCV of non-vestibular vertigo. Of the 13 patients with BPPV, 53.84% were associated with PCV (non-vestibular vertigo). The senior author observed such an association in a previous study [2].

The limitations of the study are that an MRI of the brain was not done in all patients to rule out central causes of giddiness due to financial constraints, and this study was done in a small number of patients in a single institution.

## Conclusions

This is probably the first study that attempted to classify vertigo into vestibular, central, and non-vestibular vertigo of cervicogenic type on the basis of the likely site of origin and correlate them with the vertigo syndromes of vertigo and disequilibrium and their overlaps.

Vertigo with disequilibrium is more frequent than reported previously and could be a result of origin at multiple levels in the vestibular pathway. Central causes are known to cause vertigo with disequilibrium; however, the present study concluded one of the most important causes of vertigo with disequilibrium to be PCV, a form of non-vestibular vertigo, occurring alone or in association with different etiologies of vestibular vertigo like Meniere's disease and BPPV. The occurrence of disequilibrium in all the patients of PCV emphasizes the need for the evaluation of vertigo syndromes by a team approach, with the inclusion of physiotherapy assessment as a routine part of the protocol.

The authors of the present study believe that overlooking the entity of the cervicogenic form of non-vestibular vertigo might miss the appropriate diagnosis in a significant number of patients and make them inappropriately labeled as psychogenic. As an otolaryngologist who has referrals of patients with vertigo with/without disequilibrium, knowledge of the entity of non-vestibular vertigo of cervicogenic origin becomes crucial.
